# Supplementary material for: Concerning the Role of σ-Hole in Non-Covalent Interactions: Insights from the Study of the Complexes of ArBeO with Simple Ligands
Source: Molecules. 2021 Jul 24;26(15):4477. doi: 10.3390/molecules26154477 (PMC8348141; doi:10.3390/molecules26154477)
Supplement: Supplementary file 1 [file molecules-26-04477-s001.zip › molecules-1284080-supplementary.pdf]

Concerning the Role of σ-hole in Non-Covalent Interactions: Insights from the Study of the Complexes of ArBeO with Simple Ligands

Stefano Borocci,[a,b] Felice Grandinetti,*[,a,b] and Nico Sanna[a,c]

to be published in *molecules*

---

[a] *Prof. S. Borocci, Prof. F. Grandinetti, Prof. N. Sanna*

Dipartimento per la Innovazione nei sistemi Biologici, Agroalimentari e Forestali (DIBAF), Università della Tuscia, L.go dell'Università, s.n.c., 01100 Viterbo (Italy)

E-mail: fgrandi@unitus.it

orcid.org/0000-0001-7217-9490

[b] *Prof. S. Borocci, Prof. F. Grandinetti*

Istituto per i Sistemi Biologici del CNR, Via Salaria, Km 29.500, 00015, Monterotondo, RM (Italy)

[c] *Prof. N. Sanna*

Istituto per la Scienza e Tecnologia dei Plasmi (ISTP) del CNR,Via Amendola, 122/D, 70126 Bari (Italy)

Supplementary Information

Table S1. MP2/aVTZ and CCSDT(T)/aVTZ Ar-X distances (Å) and harmonic vibrational frequencies (cm^{-1}) of the *L*Ar and *L*-ArBeO.

	**Ar-X** [a]			**Frequencies**		
		MP2	CCSD(T)		MP2	CCSD(T)
**HeAr**	Ar-He	3.5421	3.4973	σ_g	30.8	34.2
**NeAr**	Ar-Ne	3.5441	3.5178	σ_g	26.2	27.6
**ArAr**	Ar-Ar	3.7657	3.8134	σ_g	32.0	29.6
**NN-Ar**	Ar-N	3.6139	3.6498	σ_g/π	37.0, 2185.5/6.2	35.1, 2339.3/12.6
**N_2-Ar (T [b])**	Ar-CM	3.6062	3.6801	a_1/b_2	41.2, 2185.4/12.1	36.4, 2339.5/6.0
**OC-Ar**	Ar-C	3.8424	3.8424	σ_g/π	30.8, 2109.4/11.9*i*	31.1, 2144.2/7.3*i*
**CO-Ar**	Ar-O	3.4496	3.4833	σ_g/π	39.3, 2108.5/1.5	36.9, 2143.8/6.6
**FF-Ar**	Ar-F	3.1061	3.1628	σ_g/π	43.9, 998.5/37.0	39.8, 915.2/31.1
**F_2-Ar (T)**	Ar-CM	3.4263	3.4263	a_1/b_2	34.5, 1002.7/31.6	34.4, 916.4/31.6
**ClCl-Ar**	Ar-Cl	3.3886	3.4699	σ_g/π	45.2, 571.8/27.6	40.3, 541.5/23.4
**Cl_2-Ar (T)**	Ar-CM	3.6191	3.6853	a_1/b_2	43.0, 573.6/37.6	38.9, 542.1/33.2
**FCl-Ar**	Ar-Cl	3.2467	3.3251	σ_g/π	52.8, 795.5/51.0	47.6, 770.5/43.6
**ClF-Ar**	Ar-F	3.1953	3.2287	σ_g/π	38.1, 797.7/19.4	36.1, 771.7/16.9
**FH-Ar**	Ar-H	2.5109	2.5048	σ_g/π	69.6, 4099.8/196.4	71.7, 4105.7/195.2
**HF-Ar**	Ar-F	3.3392	3.3415	σ_g/π	40.7, 4119.9/70.9	40.8, 4122.6/67.7
**ClH-Ar**	Ar-H	2.6663	2.7113	σ_g/π	52.4, 3037.9/97.7	49.3, 2989.8/90.3
**HCl-Ar**	Ar-Cl	3.5919	3.6533	σ_g/π	42.6, 3041.5/78.3	38.7, 2989.5/69.2
**H_3N-Ar**	Ar-N	3.7809	3.7655	a_1	40.4, 1041.7, 3501.6	41.6, 1066.8, 3463.0
				e	17.0*i*, 1669.1, 3648.3	10.5*i*, 1672.9, 3591.4
**He-ArBeO**	Ar-He	3.2431	3.2167	σ_g/π	51.3, 269.5, 1490.4/6.8, 163.4	54.7, 272.6, 1536.4/7.4, 156.4
**Ne-ArBeO**	Ar-Ne	3.2991	3.2740	σ_g/π	36.2, 270.1, 1490.4/5.1,163.9	38.1, 273.4, 1536.5/4.8, 156.6
**Ar-ArBeO**	Ar-Ar	3.5759	3.6065	σ_g/π	38.6, 271.6, 1490.4/6.7, 164.8	36.9, 274.7, 1536.3/6.1, 157.9
**HF-ArBeO**	Ar-F	2.9032	2.9040	σ_g	89.3, 288.0, 1492.2, 4099.8	89.8, 291.3, 1536.9, 4106.3
				π	13.4, 122.9, 175.1	12.6, 118.8, 169.5
		MP2				
**NN-ArBeO**	Ar-N	3.2775		σ_g/π	57.3,277.4,1490.7,2187.7/9.7,58.0,167.2	
**N_2-ArBeO (T)**	Ar-CM	3.5420		$a_1/b_1/b_2$	38.5,269.0,1489.9,2183.6/5.6,161.4/52.5*i*, 5.3, 191.2	
**OC-ArBeO**	Ar-C	3.3811		σ_g/π	60.4, 281.2,1490.3, 2120.3/10.8, 74.4,169.7	
**CO-ArBeO**	Ar-O	3.1894		σ_g/π	54.9, 274.9,1491.6, 2104.8/8.6, 44.9, 165.1	
**FF-ArBeO**	Ar-F	3.1745		σ_g/π	30.5, 267.1, 1006.1, 1489.9/32.1*i*, 2.4*i*, 159.3	
**F_2-ArBeO (T)**	Ar-CM	3.1795		$a_1/b_1/b_2$	47.0, 273.5,999.1/7.4,163.5/4.8, 45.9, 163.7	
**ClCl-ArBeO**	Ar-Cl	3.6223		σ_g/π	22.8, 265.1, 574.9, 1489.1/29.6*i*, 2.3*i*, 158.7	
**Cl_2-ArBeO (T)**	Ar-CM	3.4276		$a_1/b_1/b_2$	50.1, 277.3, 571.6, 1490.4/7.6, 166.0/3.7*i*, 45.3, 166.2	
**ClF-ArBeO**	Ar-F	2.9614		σ_g/π	52.2, 277.4, 796.6, 1491.2/5.6, 10.0, 166.0	
**HCl-ArBeO**	Ar-Cl	3.4929		σ_g/π	44.4, 272.2, 1489.9, 3034.3/122.0*i*, 6.0, 163.5	
**H_3N-ArBeO**	Ar-N	3.1286		a_1	104.9, 300.6, 1105.4, 1492.8, 3494.5	
				e	19.6, 183.4, 209.7, 1671.4, 3633.2	

[a] X = closest atom/center-of-mass (CM) of X_2 for linear/T-shaped species. [b] T-shaped.

Data S1. Cartesian coordinates (Å) of the *L*Ar and *L*-ArBeO

HeAr
MP2/aVTZ

Ar	0.000000	0.000000	0.000000
He	3.542092	0.000000	0.000000

CCSD(T)/aVTZ

Ar	0.000000	0.000000	0.000000
He	3.497343	0.000000	0.000000

NeAr
MP2/aVTZ

Ar	0.000000	0.000000	0.000000
Ne	3.544096	0.000000	0.000000

CCSD(T)/aVTZ

Ar	0.000000	0.000000	0.000000
Ne	3.517812	0.000000	0.000000

ArAr
MP2/aVTZ

Ar	0.000000	0.000000	0.000000
Ar	3.765654	0.000000	0.000000

CCSD(T)/aVTZ

Ar	0.000000	0.000000	0.000000
Ar	3.813444	0.000000	0.000000

N_2-Ar
MP2/aVTZ

Ar	0.000000	0.000000	0.000000
N	3.613868	0.000000	0.000000
N	4.728054	0.000000	0.000000

CCSD(T)/aVTZ

Ar	0.000000	0.000000	0.000000
N	3.649789	0.000000	0.000000
N	4.753769	0.000000	0.000000

N_2-Ar (T-shaped)
MP2/aVTZ

Ar	0.000000	0.000000	0.000000
N	3.606201	0.000000	0.5570995
N	3.606201	0.000000	-0.5570995

CCSD(T)/aVTZ

Ar	0.000000	0.000000	0.000000
N	3.680109	0.000000	0.551983
N	3.680109	0.000000	-0.551983

OC-Ar
MP2/aVTZ

Ar	0.000000	0.000000	0.000000
C	3.842395	0.000000	0.000000
O	4.981386	0.000000	0.000000

CCSD(T)/aVTZ

Ar	0.000000	0.000000	0.000000
C	3.842397	0.000000	0.000000
O	4.978383	0.000000	0.000000

CO-Ar

MP2/aVTZ

Ar	0.000000	0.000000	0.000000
O	3.449593	0.000000	0.000000
C	4.588669	0.000000	0.000000

CCSD(T)/aVTZ

Ar	0.000000	0.000000	0.000000
O	3.483322	0.000000	0.000000
C	4.619335	0.000000	0.000000

F_2-Ar

MP2/aVTZ

Ar	0.000000	0.000000	0.000000
F	3.106127	0.000000	0.000000
F	4.508576	0.000000	0.000000

CCSD(T)/aVTZ

Ar	0.000000	0.000000	0.000000
F	3.162790	0.000000	0.000000
F	4.581308	0.000000	0.000000

F_2-Ar (T-shaped)

MP2/aVTZ

Ar	0.000000	0.000000	0.000000
F	3.426341	0.000000	0.700749
F	3.426341	0.000000	-0.700749

CCSD(T)/aVTZ

Ar	0.000000	0.000000	0.000000
F	3.426298	0.000000	0.709056
F	3.426298	0.000000	-0.709056

Cl_2-Ar

MP2/aVTZ

Ar	0.000000	0.000000	0.000000
Cl	3.388634	0.000000	0.000000
Cl	5.388335	0.000000	0.000000

CCSD(T)/aVTZ

Ar	0.000000	0.000000	0.000000
Cl	3.469899	0.000000	0.000000
Cl	5.489794	0.000000	0.000000

Cl_2-Ar (T-shaped)

MP2/aVTZ

Ar	0.000000	0.000000	0.000000
Cl	3.619097	0.000000	0.999162
Cl	3.619097	0.000000	-0.999162

CCSD(T)/aVTZ

Ar	0.000000	0.000000	0.000000
Cl	3.685349	0.000000	1.009552
Cl	3.685349	0.000000	-1.009552

FCl-Ar

MP2/aVTZ

Ar	0.000000	0.000000	0.000000
Cl	3.246687	0.000000	0.000000
F	4.886555	0.000000	0.000000

CCSD(T)/aVTZ

Ar	0.000000	0.000000	0.000000
Cl	3.325118	0.000000	0.000000
F	4.972285	0.000000	0.000000

ClF-Ar

MP2/aVTZ

Ar	0.000000	0.000000	0.000000
F	3.195294	0.000000	0.000000
Cl	4.834456	0.000000	0.000000

CCSD(T)/aVTZ

Ar	0.000000	0.000000	0.000000
F	3.228704	0.000000	0.000000
Cl	4.875472	0.000000	0.000000

FH-Ar

MP2/aVTZ

Ar	0.000000	0.000000	0.000000
H	2.510865	0.000000	0.000000
F	3.433739	0.000000	0.000000

CCSD(T)/aVTZ

Ar	0.000000	0.000000	0.000000
H	2.504846	0.000000	0.000000
F	3.426750	0.000000	0.000000

HF-Ar

MP2/aVTZ

Ar	0.000000	0.000000	0.000000
F	3.339206	0.000000	0.000000
H	4.261224	0.000000	0.000000

CCSD(T)/aVTZ

Ar	0.000000	0.000000	0.000000
F	3.341485	0.000000	0.000000
H	4.262585	0.000000	0.000000

ClH-Ar

MP2/aVTZ

Ar	0.000000	0.000000	0.000000
H	2.666308	0.000000	0.000000
Cl	3.941738	0.000000	0.000000

CCSD(T)/aVTZ

Ar	0.000000	0.000000	0.000000
H	2.711337	0.000000	0.000000
Cl	3.990636	0.000000	0.000000

HCl-Ar

MP2/aVTZ

Ar	0.000000	0.000000	0.000000
Cl	3.591854	0.000000	0.000000
H	4.866893	0.000000	0.000000

CCSD(T)/aVTZ

Ar	0.000000	0.000000	0.000000
Cl	3.653298	0.000000	0.000000
H	4.932325	0.000000	0.000000

H_3N-Ar

MP2/aVTZ

Ar	0.000000	0.000000	0.000000
N	3.780873	0.000000	0.000000
H	4.161841	0.000000	0.937834
H	4.161841	0.812188	-0.468917
H	4.161841	-0.812188	-0.468917

CCSD(T)/aVTZ

Ar	0.000000	0.000000	0.000000
N	3.765453	0.000000	0.000000
H	4.152642	0.000000	0.938226
H	4.152642	0.812527	-0.469113
H	4.152642	-0.812527	-0.469113

He-ArBeO

MP2/aVTZ

Ar	0.000000	0.000000	0.000000
Be	2.078054	0.000000	0.000000
O	3.429223	0.000000	0.000000
He	-3.243142	0.000000	0.000000

CCSD(T)/aVTZ

Ar	0.000000	0.000000	0.000000
Be	2.074342	0.000000	0.000000
O	3.415667	0.000000	0.000000
He	-3.216686	0.000000	0.000000

Ne-ArBeO

MP2/aVTZ

Ar	0.000000	0.000000	0.000000
Be	2.077510	0.000000	0.000000
O	3.428697	0.000000	0.000000
Ne	-3.299058	0.000000	0.000000

CCSD(T)/aVTZ

Ar	0.000000	0.000000	0.000000
Be	2.073680	0.000000	0.000000
O	3.415018	0.000000	0.000000
Ne	-3.273997	0.000000	0.000000

Ar-ArBeO

MP2/aVTZ

Ar	0.000000	0.000000	0.000000
Be	2.076147	0.000000	0.000000
O	3.427404	0.000000	0.000000
Ar	-3.575896	0.000000	0.000000

CCSD(T)/aVTZ

Ar	0.000000	0.000000	0.000000
Be	2.072500	0.000000	0.000000
O	3.413916	0.000000	0.000000
Ar	-3.606492	0.000000	0.000000

N_2-ArBeO

MP2/aVTZ

Ar	0.000000	0.000000	0.000000
Be	2.069828	0.000000	0.000000
O	3.421416	0.000000	0.000000

N	-3.277501	0.000000	0.000000
N	-4.391416	0.000000	0.000000

N_2-ArBeO (T-shaped)
MP2/aVTZ

Ar	0.000000	0.000000	0.000000
Be	2.078273	0.000000	0.000000
O	3.429536	0.000000	0.000000
N	-3.541996	0.000000	0.557212
N	-3.541996	0.000000	-0.557212

OC-ArBeO
MP2/aVTZ

Ar	0.000000	0.000000	0.000000
Be	2.065773	0.000000	0.000000
O	3.417668	0.000000	0.000000
C	-3.381107	0.000000	0.000000
O	-4.518611	0.000000	0.000000

CO-ArBeO
MP2/aVTZ

Ar	0.000000	0.000000	0.000000
Be	2.072712	0.000000	0.000000
O	3.423965	0.000000	0.000000
O	-3.189422	0.000000	0.000000
C	-4.329420	0.000000	0.000000

F_2-ArBeO
MP2/aVTZ

Ar	0.000000	0.000000	0.000000
Be	2.080132	0.000000	0.000000
O	3.431308	0.000000	0.000000
F	-3.174483	0.000000	0.000000
F	-4.574741	0.000000	0.000000

F_2-ArBeO (T-shaped)
MP2/aVTZ

Ar	0.000000	0.000000	0.000000
Be	2.073693	0.000000	0.000000
O	3.425128	0.000000	0.000000
F	-3.179484	0.000000	0.701473
F	-3.179484	0.000000	-0.701473

Cl_2-ArBeO
MP2/aVTZ

Ar	0.000000	0.000000	0.000000
Be	2.082255	0.000000	0.000000
O	3.433453	0.000000	0.000000
Cl	-3.622299	0.000000	0.000000
Cl	-5.619307	0.000000	0.000000

Cl_2-ArBeO (T-shaped)
MP2/aVTZ

Ar	0.000000	0.000000	0.000000
Be	2.070047	0.000000	0.000000

O	3.421661	0.000000	0.000000
Cl	-3.427555	0.000000	1.000297
Cl	-3.427555	0.000000	-1.000297

ClF-ArBeO

MP2/aVTZ

Ar	0.000000	0.000000	0.000000
Be	2.070024	0.000000	0.000000
O	3.421507	0.000000	0.000000
F	-2.961366	0.000000	0.000000
Cl	-4.604040	0.000000	0.000000

HF-ArBeO

MP2/aVTZ

Ar	0.000000	0.000000	0.000000
Be	2.060280	0.000000	0.000000
O	3.412135	0.000000	0.000000
F	-2.903182	0.000000	0.000000
H	-3.826560	0.000000	0.000000

CCSD(T)/aVTZ

Ar	0.000000	0.000000	0.000000
Be	2.056844	0.000000	0.000000
O	3.398998	0.000000	0.000000
F	-2.904036	0.000000	0.000000
H	-3.826362	0.000000	0.000000

HCl-ArBeO

MP2/aVTZ

Ar	0.000000	0.000000	0.000000
Be	2.075010	0.000000	0.000000
O	3.426486	0.000000	0.000000
Cl	-3.492872	0.000000	0.000000
H	-4.768685	0.000000	0.000000

H_3N-ArBeO

MP2/aVTZ

Ar	0.000000	0.000000	0.000000
Be	2.046988	0.000000	0.000000
O	3.399617	0.000000	0.000000
N	-3.128550	0.000000	0.000000
H	-3.519296	0.000000	0.934951
H	-3.519296	0.809691	-0.467475
H	-3.519296	-0.809691	-0.467475

Table S2. MP2/aVTZ and CCSD(T)/aVTZ properties of the nCov(C) bonds of the *L*Ar and *L*-ArBeO. $N(\Omega_s)$, ρ_s(ave), and H_s(ave/max/min) are, respectively, the total electronic charge (m*e*), the average electron density ($e\ a_0^{-3}$), and the average, maximum and minimum of $H(\mathbf{r})$ (hartree a_0^{-3}) over the volume Ω_s (a_0^3) enclosed by the $s(\mathbf{r})$ = 0.4 isosurface at around the BCP.

**Bond**	***L*Ar**		Ω_s	$N(\Omega_s)$	ρ_s**(ave)**	H_s**(ave/max/min)**
**He-Ar**	HeAr	MP2	0.0212	0.024	0.0011	0.00040/0.00042/0.00038
		CCSD(T)	0.0215	0.025	0.0011	0.00040/0.00042/0.00038
**Ne-Ar**	NeAr	MP2	0.0316	0.063	0.0020	0.00059/0.00061/0.00057
		CCSD(T)	0.0313	0.062	0.0020	0.00058/0.00060/0.00057
**Ar-Ar**	ArAr	MP2	0.0948	0.27	0.0029	0.00074/0.00078/0.00069
		CCSD(T)	0.0946	0.27	0.0029	0.00073/0.00077/0.00068
**N-Ar**	N_2-Ar	MP2	0.0788	0.22	0.0028	0.00076/0.00080/0.00071
		CCSD(T)	0.0780	0.22	0.0028	0.00076/0.00080/0.00071
	N_2-Ar (T [a])	MP2	0.1570	0.46	0.0029	0.00075/0.00082/0.00067
		CCSD(T)	0.1525	0.45	0.0030	0.00075/0.00081/0.00068
**C-Ar**	OC-Ar	MP2	0.0914	0.21	0.0023	0.00060/0.00063/0.00056
		CCSD(T)	0.0901	0.21	0.0023	0.00059/0.00062/0.00055
**O-Ar**	CO-Ar	MP2	0.0736	0.24	0.0032	0.00086/0.00091/0.00080
		CCSD(T)	0.0729	0.23	0.0032	0.00086/0.00091/0.00080
**F-Ar**	F_2-Ar	MP2	0.0966	0.46	0.0047	0.00154/0.00176/0.00137
		CCSD(T)	0.0952	0.45	0.0047	0.00154/0.00176/0.00136
	F_2-Ar (T)	MP2	0.2052	0.59	0.0029	0.00068/0.00076/0.00058
		CCSD(T)	0.2024	0.57	0.0028	0.00067/0.00075/0.00058
**Cl-Ar**	Cl_2-Ar	MP2	0.1687	0.90	0.0053	0.00153/0.00165/0.00139
		CCSD(T)	0.1651	0.87	0.0053	0.00153/0.00164/0.00139
	Cl_2-Ar (T)	MP2	0.4444	1.56	0.0035	0.00088/0.00095/0.00076
		CCSD(T)	0.4398	1.53	0.0035	0.00087/0.00094/0.00076
**Cl-Ar**	FCl-Ar	MP2	0.2167	1.44	0.0067	0.00184/0.00199/0.00165
		CCSD(T)	0.2119	1.41	0.0066	0.00184/0.00198/0.00164
**F-Ar**	ClF-Ar	MP2	0.0866	0.38	0.0043	0.00121/0.00132/0.00111
		CCSD(T)	0.0852	0.37	0.0043	0.00121/0.00131/0.00111
**H-Ar**	FH-Ar	MP2	0.0777	0.65	0.0084	0.00148/0.00158/0.00134
		CCSD(T)	0.0765	0.64	0.0084	0.00149/0.00158/0.00136
**F-Ar**	HF-Ar	MP2	0.0700	0.23	0.0032	0.00092/0.00097/0.00086
		CCSD(T)	0.0687	0.22	0.0032	0.00092/0.00097/0.00086
**H-Ar**	ClH-Ar	MP2	0.0922	0.64	0.0069	0.00129/0.00140/0.00123
		CCSD(T)	0.0921	0.64	0.0070	0.00130/0.00141/0.00123
**Cl-Ar**	HCl-Ar	MP2	0.1268	0.46	0.0037	0.00112/0.00120/0.00102
		CCSD(T)	0.1263	0.46	0.0036	0.00111/0.00119/0.00102
**N-Ar**	H_3N-Ar	MP2	0.1113	0.31	0.0028	0.00055/0.00063/0.00051
		CCSD(T)	0.1073	0.30	0.0028	0.00055/0.00063/0.00051

[a] T-shaped.

Table S3. Values of the MEP of *L* (kcal mol^{-1}) at the points of minimum ($V_{S,Min}$) or maximum ($V_{S,Max}$) calculated at the experimental (Exp. [a]), and at the CCSD(T)/aVTZ and MP2/aVTZ optimized geometries (Å and °). The mapped isodensity surfaces are 0.0005, 0.0010, 0.0015, and 0.0020 $e\ a_0^{-3}$.

L	MEP points	*R*/*θ*	Level	0.0005	0.0010	0.0015	0.0020
**N_2**	$V_{S,Min}$(N)/$V_{S,Max}$(perp)	1.0977 (Exp)	CCSD(T)/aVTZ	-7.26/5.40	-8.46/7.40	-9.08/9.16	-9.44/10.79
		1.1040 [CCSD(T)/aVTZ]	CCSD(T)/aVTZ	-7.21/5.29	-8.38/7.27	-8.99/9.01	-9.33/10.63
		1.1141 (MP2/aVTZ)	CCSD(T)/aVTZ	-7.06 /5.17	-8.20/7.11	-8.78/8.84	-9.10/10.44
			MP2/aVTZ	-7.34/5.72	-8.53/7.82	-9.15/9.66	-9.49/11.37
**CO**	$V_{S,Min}$(C)/$V_{S,Min}$(O)	1.1283 (Exp)	CCSD(T)/aVTZ	-10.80/-5.25	-12.83/-5.85	-13.99/-6.04	-14.75/-6.05
		1.1360 [CCSD(T)/aVTZ]	CCSD(T)/aVTZ	-10.51/-5.41	-12.50/-6.02	-13.62/-6.21	-14.36/-6.22
		1.1390 (MP2/aVTZ)	CCSD(T)/aVTZ	-10.40/-5.47	-12.36/-6.08	-13.48/-6.28	-14.21/-6.28
			MP2/aVTZ	-11.87/-3.75	-14.04/-4.12	-15.29/-4.17	-16.08/-4.04
**F_2**	$V_{S,Max}$(F)/$V_{S,Max}$(perp)	1.4119 (Exp)	CCSD(T)/aVTZ	12.44/-0.23	16.36/0.90	19.54/2.10	22.39/3.33
		1.4181 [CCSD(T)/aVTZ]	CCSD(T)/aVTZ	12.55/-0.32	16.50/0.80	19.68/1.98	22.55/3.21
		1.4014 (MP2/aVTZ)	CCSD(T)/aVTZ	12.25/-0.09	16.14/1.07	19.29/2.30	22.11/3.56
			MP2/aVTZ	12.59/-0.34	16.57/0.76	19.80/1.93	22.67/3.14
**Cl_2**	$V_{S,Max}$(Cl)/$V_{S,Max}$(perp)	1.9879 (Exp)	CCSD(T)/aVTZ	19.02/-0.28	25.02/1.73	29.54/3.75	33.63/5.71
		2.0194 [CCSD(T)/aVTZ]	CCSD(T)/aVTZ	19.32/-0.61	25.34/1.32	29.97/3.27	34.0/5.20
		1.9987 (MP2/aVTZ)	CCSD(T)/aVTZ	19.11/-0.40	25.13/1.59	29.69/3.58	33.74/5.52
			MP2/aVTZ	19.38/-0.64	25.50/1.26	30.14/3.19	34.24/5.09
**ClF**	$V_{S,Max}$(Cl)/$V_{S,Max}$(F)	1.6281 (Exp [b])	CCSD(T)/aVTZ	31.56/-3.25	39.89/-2.23	46.33/-1.16	51.33/-0.05
		1.6463 [CCSD(T)/aVTZ]	CCSD(T)/aVTZ	32.00/-3.37	40.38/-2.34	46.85/-1.21	51.88/-0.12
		1.6385 (MP2/aVTZ)	CCSD(T)/aVTZ	31.82/-3.32	40.17/-2.30	46.63/-1.19	51.65/-0.09
			MP2/aVTZ	32.38/-2.97	40.94/-1.86	47.56/-0.68	52.69/0.47
**HF**	$V_{S,Max}$(H)/$V_{S,Max}$(F)	0.9168 (Exp)	CCSD(T)/aVTZ	55.15/-17.92	68.19/-18.97	77.96/-19.38	85.96/-19.45
		0.9210 [CCSD(T)/aVTZ]	CCSD(T)/aVTZ	55.18/-17.94	68.23/-18.98	78.03/-19.39	86.08/-19.46
		0.9218 (MP2/aVTZ)	CCSD(T)/aVTZ	55.18/-17.94	68.24/-18.99	78.04/-19.39	86.10/-19.46
			MP2/aVTZ	55.56/-17.87	68.78/-18.91	78.71/-19.28	86.90/-19.36
**HCl**	$V_{S,Max}$(H)/$V_{S,Max}$(Cl)	1.2746 (Exp)	CCSD(T)/aVTZ	35.55/5.66	43.92/9.26	50.30/12.28	55.95/14.93
		1.2789 [CCSD(T)]	CCSD(T)/aVTZ	35.60/5.66	44.0/9.27	50.37/12.28	56.07/14.94
		1.2747 (MP2/aVTZ)	CCSD(T)/aVTZ	35.55/5.66	43.93/9.26	50.30/12.28	55.95/14.93
			MP2/aVTZ	36.70/5.39	45.38/9.00	52.08/11.93	57.90/14.63
**NH_3**	$V_{S,Min}$(N)	1.0124/106.67 (Exp [c])	CCSD(T)/aVTZ	-32.88	-38.64	-42.28	-44.93
		1.0149/106.40 [CCSD(T)/aVTZ]	CCSD(T)/aVTZ	-31.59	-37.30	-41.17	-44.10
		1.0121/106.77 (MP2/aVTZ)	CCSD(T)/aVTZ	-31.53	-37.24	-41.12	-44.06
			MP2/aVTZ	-31.52	-37.25	-41.13	-44.11

[a] Unless stated otherwise, all experimental values are from Ref. [1]. [b] Ref. [2]. [c] Ref. [3].

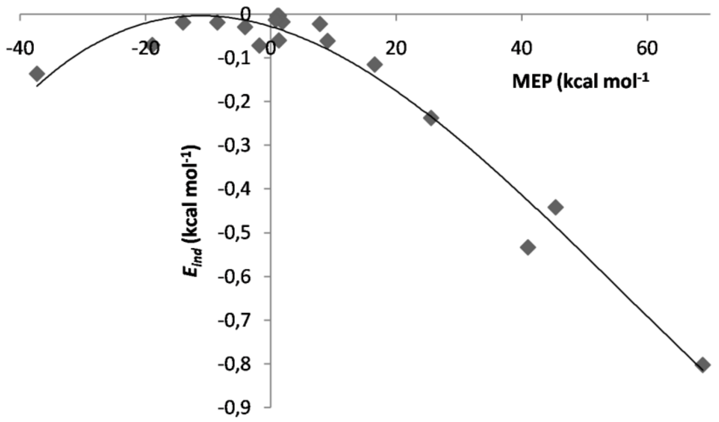

Figure S1. E_{ind} of the *L*Ar vs. MEP at the $V_{S,max}$/$V_{S,min}$ of *L*.

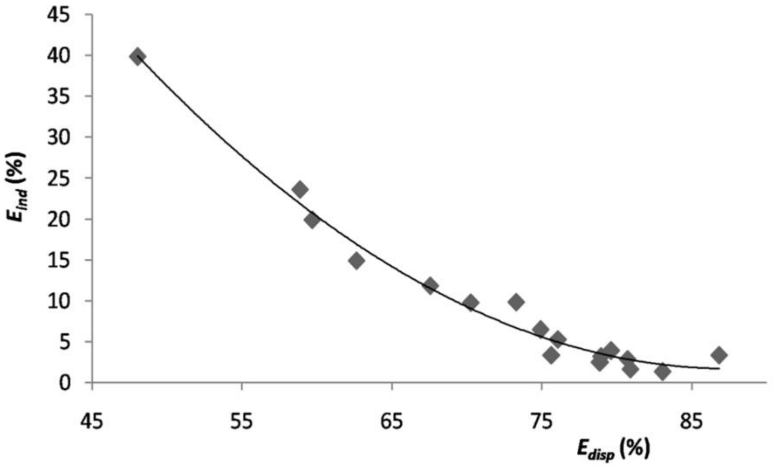

Figure S2. E_{ind}(%) vs. E_{disp}(%) of the *L*Ar.

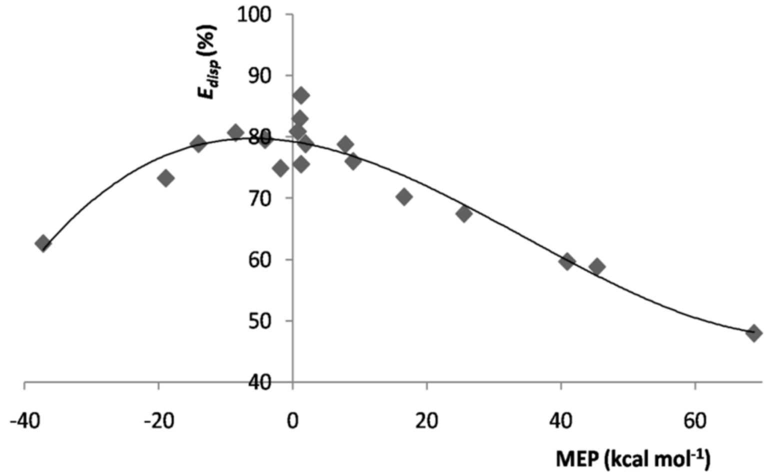

Figure S3. E_{disp}(%) of the *L*Ar vs. MEP at the $V_{S,max}$/$V_{S,min}$ of *L*.

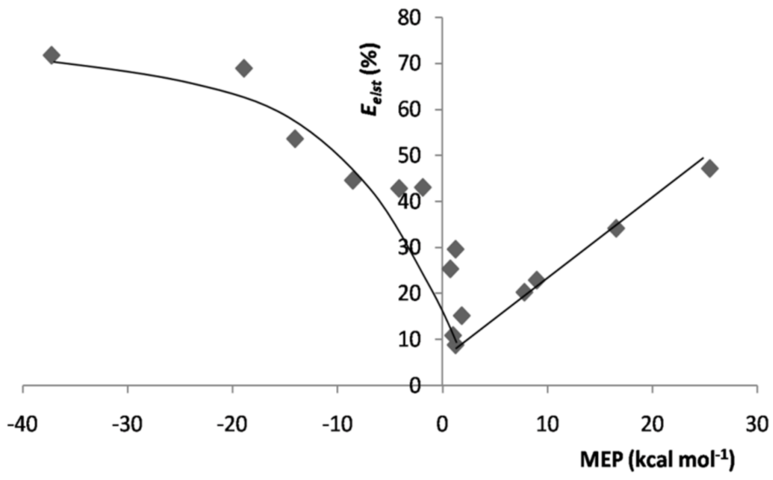

Figure S4. E_{elst}(%) of the L-ArBeO vs. MEP at the $V_{S,max}/V_{S,min}$ of L.

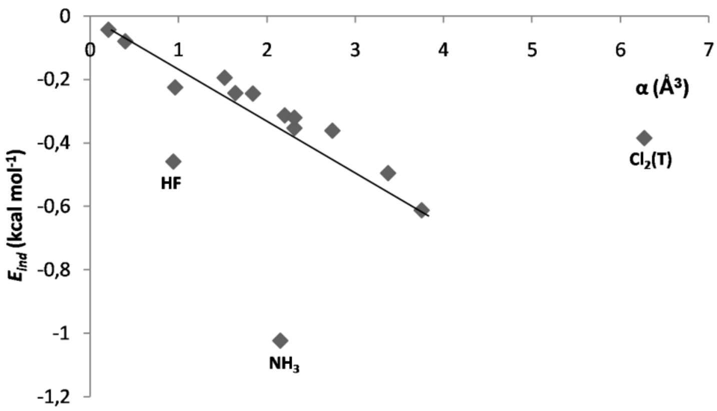

Figure S5. E_{ind} of the L-ArBeO vs. polarizability α of L.

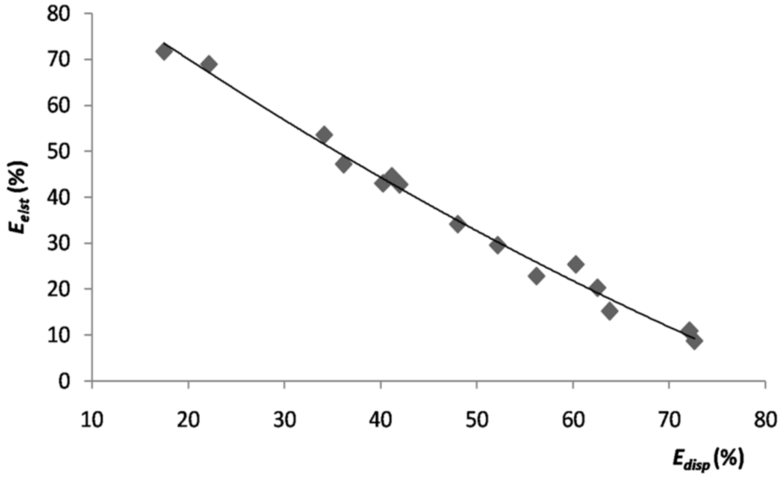

Figure S6. E_{elst}(%) vs. E_{disp}(%) of the L-ArBeO.

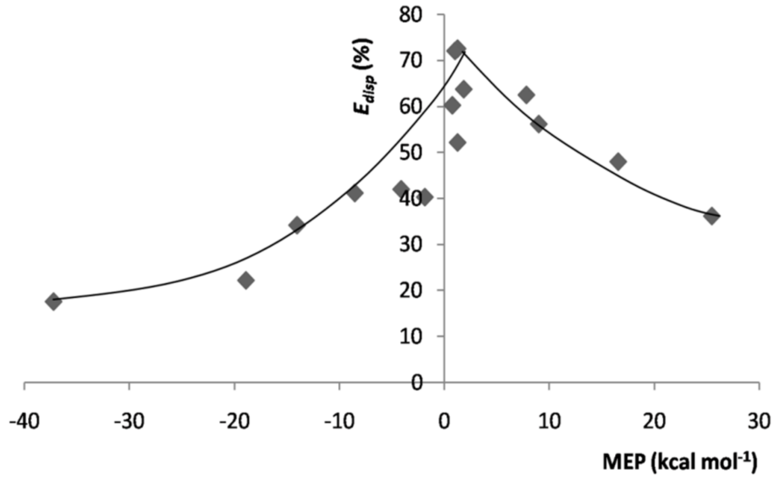

Figure S7. E_{disp}(%) of the *L*-ArBeO vs. MEP at the $V_{S,max}$/$V_{S,min}$ of *L*.

References

1. Linstrom P.J.; Mallard, W.G. Eds., *NIST Chemistry WebBook, NIST Standard Reference Database Number 69*, National Institute of Standards and Technology, Gaithersburg MD, 20899, https://doi.org/10.18434/T4D303, (retrieved December 15, 2020).
2. Gilbert, D.A.; Roberts, A.; Griswold, P.A. Nuclear and Molecular Information from the Microwave Spectrum of FCl. *Phys. Rev.* **1949**, *76*, 1723.
3. Diercksen, G.H.; Sadlej, A.J. Finite-field Many-body Perturbation Theory IX. Electric Properties of Ammonia. *Mol. Phys.* **1986**, *57*, 509-528.
